# KAP1 stabilizes MYCN mRNA and promotes neuroblastoma tumorigenicity by protecting the RNA m^6^A reader YTHDC1 protein degradation

**DOI:** 10.1186/s13046-024-03040-9

**Published:** 2024-05-14

**Authors:** Yi Yang, Yingwen Zhang, Guoyu Chen, Bowen Sun, Fei Luo, Yijin Gao, Haizhong Feng, Yanxin Li

**Affiliations:** 1 Pediatric Translational Medicine Institute, Department of Hematology & Oncology, Shanghai Children’s Medical Center, Shanghai Jiao Tong University School of Medicine, National Health Committee Key Laboratory of Pediatric Hematology & Oncology, Shanghai, 200127 China; 2 State Key Laboratory of Systems Medicine for Cancer, Renji-Med X Clinical Stem Cell Research Center, Ren Ji Hospital, Shanghai Cancer Institute, Shanghai Jiao Tong University School of Medicine, Shanghai, 200127 China

**Keywords:** MYCN, Neuroblastoma, KAP1, YTHDC1, METTL3, m^6^A, mRNA stability

## Abstract

**Background:**

Neuroblastoma (NB) patients with amplified MYCN often face a grim prognosis and are resistant to existing therapies, yet MYCN protein is considered undruggable. KAP1 (also named TRIM28) plays a crucial role in multiple biological activities. This study aimed to investigate the relationship between KAP1 and MYCN in NB.

**Methods:**

Transcriptome analyses and luciferase reporter assay identified that KAP1 was a downstream target of MYCN. The effects of KAP1 on cancer cell proliferation and colony formation were explored using the loss-of-function assays in vitro and in vivo. RNA stability detection was used to examine the influence of KAP1 on MYCN expression. The mechanisms of KAP1 to maintain MYCN mRNA stabilization were mainly investigated by mass spectrum, immunoprecipitation, RIP-qPCR, and western blotting. In addition, a xenograft mouse model was used to reveal the antitumor effect of STM2457 on NB.

**Results:**

Here we identified KAP1 as a critical regulator of MYCN mRNA stability by protecting the RNA N^6^-methyladenosine (m^6^A) reader YTHDC1 protein degradation. KAP1 was highly expressed in clinical MYCN-amplified NB and was upregulated by MYCN. Reciprocally, KAP1 knockdown reduced MYCN mRNA stability and inhibited MYCN-amplified NB progression. Mechanistically, KAP1 regulated the stability of MYCN mRNA in an m^6^A-dependent manner. KAP1 formed a complex with YTHDC1 and RNA m^6^A writer METTL3 to regulate m^6^A-modified MYCN mRNA stability. KAP1 depletion decreased YTHDC1 protein stability and promoted MYCN mRNA degradation. Inhibiting MYCN mRNA m^6^A modification synergized with chemotherapy to restrain tumor progression in MYCN-amplified NB.

**Conclusions:**

Our research demonstrates that KAP1, transcriptionally activated by MYCN, forms a complex with YTHDC1 and METTL3, which in turn maintain the stabilization of MYCN mRNA in an m^6^A-dependent manner. Targeting m^6^A modification by STM2457, a small-molecule inhibitor of METTL3, could downregulate MYCN expression and attenuate tumor proliferation. This finding provides a new alternative putative therapeutic strategy for MYCN-amplified NB.

**Supplementary Information:**

The online version contains supplementary material available at 10.1186/s13046-024-03040-9.

## Background

Neuroblastoma (NB) is the most common extracranial solid tumor in childhood, accounting for 15% of all pediatric cancer fatalities [1]. The prognostic trajectory of NB patients exhibits significant variability, encompassing a spectrum from spontaneous regression to aggressive disease progression that could lead to mortality [2]. MYCN amplification is one characteristic feature of high-risk NB [3, 4]. However, MYCN protein remains undruggable in clinical practice up till now [5–8]. Thus, a new alternative putative therapeutic strategy is urgent for MYCN-amplified NB.

MYCN is a transcriptional factor from the MYC oncogene family. Using an shRNA screen of genes, MYCN protein is shown to be stabilized by Aurora A in a kinase-independent fashion involving protein-protein interaction [7]. CD532, a conformation-disrupting inhibitor of Aurora A, could cause the loss of MYCN and extend the survival of animals bearing MYCN-amplified NB xenografts [8]. The transcription of MYCN is blocked by bromodomain inhibitor JQ1 [9]. Through the application of functional genomics, possible druggable targets have been discovered [10, 11]. Unfortunately, the current regulatory measures for MYCN transcription have proven ineffective.

KAP1, also known as TRIM28, is a member of the tripartite-motif (TRIM) family. It is highly expressed in various cancer types and contributes to tumor initiation, progress, and therapy resistance [12, 13]. KAP1 has E3 ubiquitin ligase activity to ubiquitin AMPK [14] and p53 [15] and E3 SUMO ligase activity to SUMOylate CDK9 [16] and PCNA [17]. As a versatile nuclear scaffold protein, KAP1 also participates in or coordinates the assembly of various protein complexes. For example, KAP1 interacts with SIRT3 and Lamin B1 to co-participate in stabilizing heterochromatin [18]. KAP1-SETDB1 complex inactivates the expression of endogenous retroelements and sustains genome stability [19, 20]. KAP1 directly associates with RNA polymerase II and regulates its promoter-proximal pausing and pause release [21, 22]. Recently, KAP1 is also revealed to indirectly bind to RNA and METTL3, an enzyme mediating the N6-methyladenosine (m^6^A) methylation of mRNA [23, 24], yet it is still unknown whether KAP1 regulates the stability of m^6^A-modified RNA.

Here we identify KAP1 as an independent prognostic factor of NB. As an oncoprotein, it sustains the stabilization of MYCN mRNA and contributes to NB progression. Mechanistically, KAP1 forms a complex with nuclear m^6^A reader YTHDC1 and m^6^A writer METTL3 to mediate the stability of m^6^A-modified MYCN mRNA. Thus, the destabilization of MYCN mRNA by targeting KAP1 and inhibiting METTL3 activity represents an alternative therapeutic strategy for MYCN amplified NB.

## Methods

### Cell lines and culture

SK-N-BE(2), SK-N-SH, SY-5Y, and HEK293T cells were from the American Type Culture Collection (ATCC), and CHP134 cells were from the European Collection of Authenticated Cell Cultures (ECACC). SK-N-BE(2), SK-N-SH, SY-5Y, and HEK293T cells were cultured in Dulbecco’s Modified Eagle’s Medium (DMEM) supplemented with 10% fetal bovine serum (FBS), 1% penicillin and streptomycin. CHP134 cells were cultured in Roswell Park Memorial Institute (RPMI) 1640 Medium supplemented with 10% FBS, and 1% penicillin and streptomycin. All cells were maintained in a humidified atmosphere containing 5% CO_2_ at 37 °C. All cells were tested negatively for mycoplasma contamination.

### Plasmids

The complementary sense and antisense oligonucleotides encoding shRNAs or sgRNAs targeting KAP1, YTHDC1, METTL3, or MYCN were synthesized, annealed, and cloned into pLKO.1 or lentiCRISPRv2 vector. The related sequences of shRNAs and sgRNAs were shown in Table S1. METTL3 and MYCN coding sequences were amplified from SK-N-BE(2) cells by PCR and verified by sequencing. Then they were cloned into pGV287 Vector (GeneChem, Shanghai) as we previously described [25]. pEnCMV-KAP1–3 × Flag-SV40-Neo and pEnCMV-YTHDC1–3 × Flag-SV40-Neo plasmid was purchased from Wuhan Miaolingbio Inc. (Hubei, China). YTHDC1 mutation plasmid was generated using a site-directed mutagenesis kit (Invitrogen) following the manufacturer’s protocol.

### Cell transfection and lentivirus infection

For transient transfection, cells were transfected using Hieff Trans® Liposomal Transfection Reagent (Yeason). For lentivirus production, related plasmids were co-transfected with packaging vectors psPAX2 and pMD2.G into HEK293T cells using Hieff Trans® Liposomal Transfection Reagent (Yeason). Infectious lentivirus particles were harvested at 48 h after transfection, filtered through 0.45 μm PVDF filters, and then transduced into cells.

### Clinical specimens

NB tissues were obtained from patients who had undergone surgery at the Shanghai Children’s Medical Center. In all, 12 NB tissue samples for protein extraction were freshly frozen in dry ice and stored at − 80 °C until used. This study was approved by the Institutional Review Board (IRB) of Shanghai Children’s Medical Center affiliated to Shanghai Jiao Tong University School of Medicine (SCMCIRB-K2023093–1), and informed consent was obtained from all participants.

### RNA extraction and qRT-PCR

Total RNA was isolated from cells using TRIzol according to the manufacturer’s instructions. Reverse transcription was performed using the Reverse Transcription Kit (Yeasen). qRT–PCR was performed using the SYBR Green Master Mix (Yeasen). Primers were listed in Table S2. Results were analyzed using the 2^−(ΔΔCt)^ method.

### Western blotting (WB)

WB was performed as previously described [26]. Antibodies used were as follows: anti-KAP1 (1:1000, 15,202–1-AP, Proteintech), anti-YTHDC1 (1:1000, 29,441–1-AP, Proteintech), anti-METTL3 (1:1000, 15,073–1-AP, Proteintech), anti-MYCN (1:1000, 84,406, Cell Signaling Technology), anti-Ub (1:1000, 3936, Cell Signaling Technology), anti-β-actin (1:1000, 3700, Cell Signaling Technology) and anti-GAPDH (1:50000, 60,004–1-Ig, Proteintech).

### Cell proliferation assay

Cell proliferation was measured as previously described [27]. Briefly, stable cell lines were seeded at a suitable density. Then, cell numbers were calculated using an automated cell counter every day.

### Subcutaneous xenograft experiments

All animal experiments were approved by the Ethics Committee for Laboratory Animals of Shanghai Children’s Medical Center (SCMC-LAWEC-2023-007). To assess the effects of KAP1 knockdown, SK-N-BE(2) cells (5 × 10^6^) with or without KAP1 shRNAs were injected subcutaneously into six-week-old NOD/SCID female mice. To assess the efficacy of STM2457 in NB treatment, intraperitoneal injection for 12 days with vehicle, VCR (0.5 mg/kg, 1 time per 4 days), or VCR (0.5 mg/kg, 1 time per 4 days) + STM2457 (50 mg/kg, 1 time per day) was initiated when tumors reached an average size of 150 mm^3^. Tumor growth and volume were measured every other day. At the end of feeding, mice were sacrificed with the tumors removed for histology analysis. The tumor weight and volume were recorded.

### Immunohistochemistry (IHC) and hematoxylin & eosin (H&E) staining

Formalin-fixed paraffin-embedded sections underwent H&E staining or were subjected to immunostaining using anti-KAP1 (1:200, 15,202–1-AP, Proteintech), anti-Ki-67 (1:100, GT209429, Gene Tech), or anti-MYCN (1:200, 84,406, Cell Signaling Technology) antibodies, following a standard IHC protocol.

### Co-immunoprecipitation (co-IP)

Co-IP assay was performed as previously described [27]. In brief, beads pre-coated with 5 μg antibodies against the target protein or IgG were incubated with sufficient cell lysates at 4 °C overnight. Then, the beads containing immunoprecipitated protein were lysed in SDS lysis buffer. Finally, the interested protein was tested by standard WB.

### RNA immunoprecipitation (RIP)

RIP assay was carried out in cells using Magna RIP Kit (Millipore) following the manufacturer’s instructions. In brief, magnetic beads pre-coated with 5 μg antibodies against the target protein or IgG were incubated with sufficient cell lysates at 4 °C overnight. Then, the beads containing immunoprecipitated RNA-protein complex were treated with proteinase K to remove proteins. Finally, interested RNA was purified by TRIzol methods and detected by qRT-PCR with normalization to input.

### Luciferase reporter assay

KAP1 promoter was amplified from the genomic DNA of SK-N-BE(2) cells and then cloned into pGL3-control vectors comprised of Firefly luciferase (F-luc). The mutant reporter plasmids were generated using a site-directed mutagenesis kit (Invitrogen) following the manufacturer’s protocol. HEK293T cells were seeded into a 24-well plate followed by co-transfection of wild-type or mutational KAP1 reporter plasmids and pRL-TK plasmids (Renilla luciferase reporter vector) with or without MYCN expression. After 48 h, cells were harvested to access the luciferase activity using Dual-Glo® Luciferase system (Promega) with normalization to pRL-TK. Each group was conducted in triplicate.

### Protein ubiquitination and stability assays

Protein ubiquitination assay was performed as previously described [27]. In brief, cells were pre-treated with 10 μM MG132 for 6 h before being harvested. Then, the expression level and ubiquitination status of YTHDC1 protein were determined by WB. To evaluate protein stability, cells were pre-treated with 50 μg/ml cycloheximide (CHX) during indicated times.

### RNA stability

To evaluate RNA stability, cells were treated with 5 μg/ml Actinomycin D during indicated times. Then, total RNA was isolated and the RNA expression of MYCN was determined by qPCR.

### Statistics

All statistical analyses were performed with GraphPad Prism 8.0. Statistical comparisons were performed by unpaired Student’s *t*-test, one-way ANOVA, or log-rank (Mantel-Cox) test. A value of *P* < 0.05 was considered statistically significant.

## Results

### KAP1 is upregulated by MYCN in MYCN-amplified neuroblastoma

**Fig. 1:**
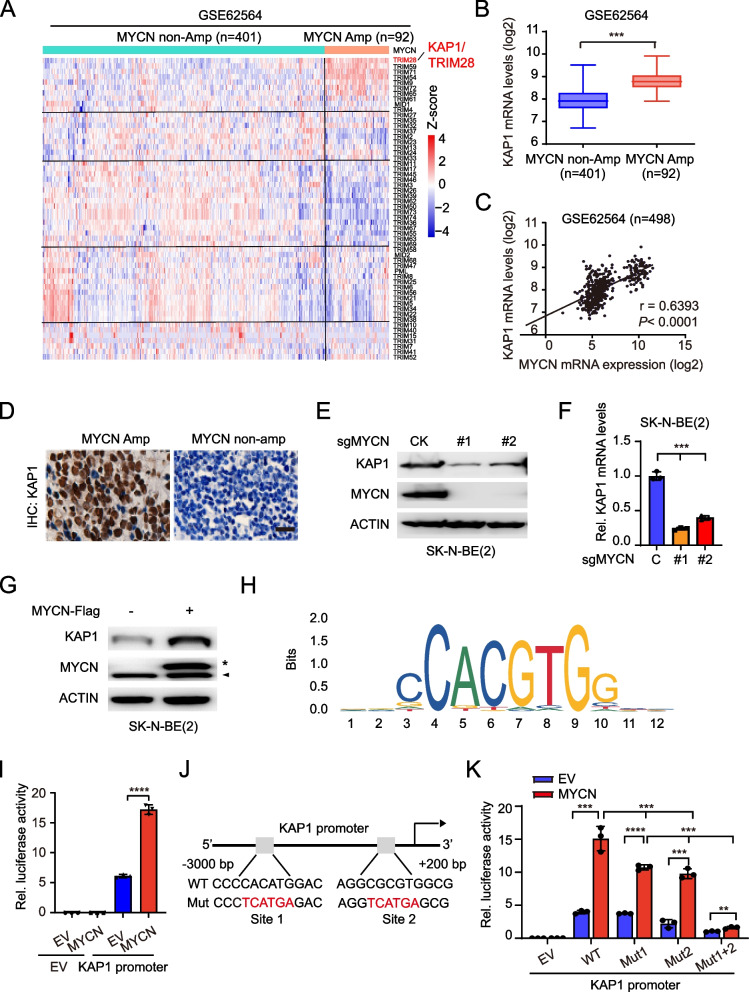
KAP1 is upregulated in MYCN-amplified neuroblastoma. **A** Heatmap of TRIM family gene expression in NB from gene expression omnibus (GEO, GSE62564). KAP1 was marked by increased font sizes and red color. **B** The relative expression of KAP1 in cohorts grouped by MYCN amplification or not. **C** Correlation between KAP1 and MYCN expression in tumors from NB patients. **D** Representative images of IHC staining of KAP1 in NB samples with MYCN amplification or not. Scale bars: 50 μm. **E** Effects of MYCN knockout (KO) on the protein levels of KAP1 in SK-N-BE(2) cells. **F** Effects of MYCN KO on the mRNA levels of KAP1 in SK-N-BE(2) cells. **G** Effects of MYCN overexpression on the protein levels of KAP1 in SK-N-BE(2) cells. *, Flag-tagged MYCN. ^▲^, endogenous MYCN. **H** Potential binding sites of KAP1 promoter with MYCN protein. **I** The relative luciferase activity of KAP1 promotor plasmid in HEK293T cells expressing vector or MYCN. Firefly luciferase activity was normalized to Renilla luciferase activity. **J** Mutation of potential binding sites of KAP1 promoter with MYCN protein. **K** The relative luciferase activity of KAP1 promotor plasmid and its mutant in HEK293T cells expressing vector or MYCN. Firefly luciferase activity was normalized to Renilla activity. Data are representative of three independent experiments in F, I and K. Error bars, ± SD. ** *P* < 0.01, *** *P* < 0.001, **** *P* < 0.0001, by unpaired two-tailed *t*-test (B, I and K), by one-way ANOVA (F and K)

To identify potential targets for treating MYCN-amplified NB, we obtained an RNA-sequencing dataset (GSE62564) from Gene Expression Omnibus (GEO) and analyzed the gene expression of the TRIM family. KAP1, also known as TRIM28, exhibited elevated expression levels in MYCN-amplified samples compared to non-MYCN amplified tumors (Fig. 1A and B). The correlation analysis revealed a strong positive relationship between the gene expression of KAP1 and MYCN (Fig. 1C). Immunohistochemistry (IHC) staining results also supported the finding that MYCN-amplified tumors exhibited higher levels of KAP1 protein expression compared to non-MYCN amplified samples (Fig. 1D).

To assess whether KAP1 was a downstream effector of MYCN, we knocked out MYCN using two different sgRNAs, sgMYCN#1 and sgMYCN#2 in SK-N-BE(2) NB cells with MYCN amplification. MYCN knockout (KO) markedly reduced KAP1 protein and mRNA expression (Fig. 1E and F). Consistently, MYCN overexpression upregulated KAP1 protein levels (Fig. 1G). These data indicates that the expression of KAP1 gene is primarily controlled by MYCN at the level of transcription.

Next, we searched the promoter sequence data of KAP1 in UCSC Genome Browser (https://genome.ucsc.edu) and found significant MYCN binding near KAP1 transcription start sites (TSS, Fig. 1H) using JASPAR database (https://jaspar.elixir.no/). To further confirm that MYCN upregulated KAP1, we performed a luciferase-based gene reporter assay and found that forced expression of MYCN increased the activity of wildtype (WT) KAP1 promoter (Fig. 1I). Mutation of either potential binding site 1 (MUT1) or 2 (MUT2) weakly reduced MYCN-induced KAP1 promoter activity, but the combination of two mutation sites (MUT1 + 2) decreased it significantly (Fig. 1J and K). Taken together, these data demonstrate that KAP1 is highly expressed in MYCN-amplified NB and directly upregulated by MYCN.

### Inhibiting KAP1, a poor prognostic factor of neuroblastoma, reduces tumorigenicity

**Fig. 2:**
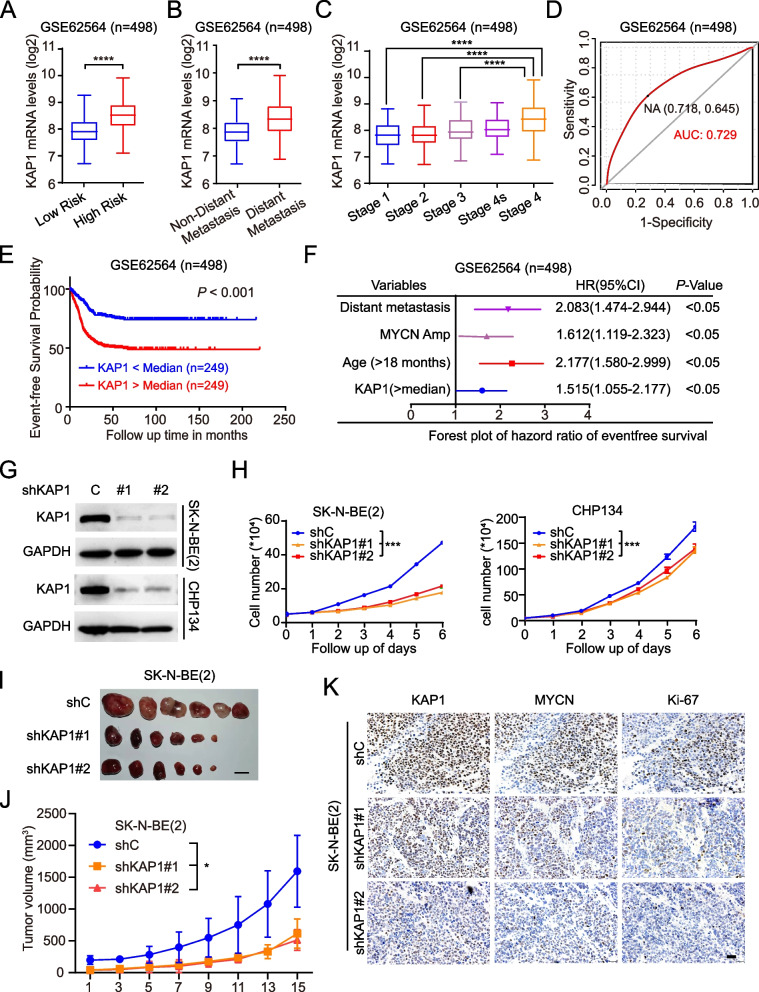
High KAP1 level predicts poor patient prognosis in neuroblastoma. **A** The relative expression of KAP1 in cohorts grouped by risk stratification. **B** The relative expression of KAP1 in cohorts grouped by distant metastasis or not. **C** The relative expression of KAP1 in cohorts grouped by INSS stages. **D** ROC curve assessment of the prognostic ability of KAP1 expression in NB. **E** Kaplan-Meier survival analysis of NB patients with different levels of KAP1 expression. **F** Cox regression analysis of the prognostic ability of KAP1 expression in NB. **G** Effects of KAP1 knockdown (KD) on the protein levels of KAP1 in SK-N-BE(2) and CHP134 cells. **H** Effects of KAP1 KD on cell proliferation in SK-N-BE(2) and CHP134 cells. **I** Images of subcutaneous xenograft tumors at the endpoints formed by SK-N-BE(2) cells with modified KAP1 expression. Scale bars: 1 cm. **J** Tumor growth curves recorded in the xenograft mice. **K** Representative images of IHC staining of KAP1, MYCN and Ki-67 in the xenograft mice. Scale bars: 50 μm. Data are representative of three independent experiments in H. Error bars, ± SD. * *P* < 0.05, *** *P* < 0.001, **** *P* < 0.0001, by unpaired two-tailed *t*-test (A, B, and C), by one-way ANOVA (H and J), by log-rank test (E)

To assess the clinical significance of KAP1 in NB, we examined KAP1 mRNA levels in cohorts grouped by different clinical factors. KAP1 levels were increased according to tumor aggressiveness (Fig. 2A-C). Specifically, the expression of KAP1 was significantly higher in cohorts belonging to the high-risk group or those who developed distant metastasis (Fig. 2A and B). We also analyzed patient survival and KAP1 gene expression from the GEO dataset GSE62564 and found that the expression levels of KAP1 were closely correlated with clinical prognosis in NB (Fig. 2D). Kaplan-Meier estimate demonstrated that elevated KAP1 levels were strongly linked to decreased event-free survival (Fig. 2E). Cox regression analysis revealed that KAP1 was an independent prognostic biomarker in NB (Fig. 2F). Our findings indicate that KAP1 is a negative prognostic marker for NB.

Evidence indicates that KAP1 and MDM2 work together to facilitate the ubiquitination and degradation of p53, while reducing KAP1 levels increases p53 transcriptional activity and leads to higher rates of cell apoptosis [15]. To minimize the p53 effect regulated by KAP1, we chose SK-N-BE(2) cell line with TP53 mutation and CHP134 cell line with TP53 wildtype in the following assays. We knocked down KAP1 using two different shRNAs, shKAP1#1 and shKAP1#2 (Fig. 2G), and found that KAP1 knockdown (KD) significantly decreased cell proliferation (Fig. 2H) and colony formation (Fig. S1A and S1B). Subcutaneous xenografts also indicated that KAP1 KD markedly reduced tumor growth (Fig. 2I and J). Furthermore, mice tumors were isolated and analyzed by IHC staining. Interestingly, KAP1 KD not only reduced the protein expression of cell proliferation marker Ki-67 but also core oncogene MYCN (Fig. 2K). In all, these data support KAP1 as an oncogene in NB.

### KAP1 regulates MYCN mRNA stability

**Fig. 3:**
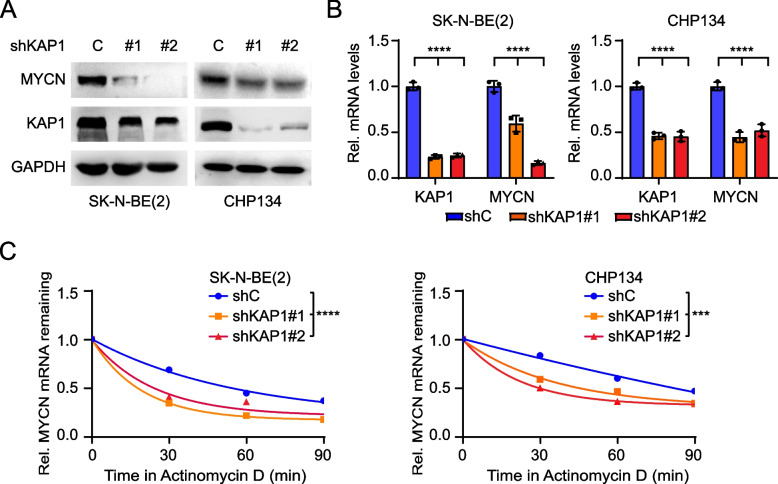
KAP1 regulates MYCN mRNA stability. **A** Effects of KAP1 knockdown (KD) on the protein levels of MYCN in SK-N-BE(2) and CHP134 cells. **B** Effects of KAP1 KD on the mRNA levels of MYCN in SK-N-BE(2) and CHP134 cells. **C** Effects of KAP1 KD on MYCN mRNA degradation in SK-N-BE(2) and CHP134 cells. Cells were treated with Actinomycin D at indicated times. Data are representative of three independent experiments in B and C. Error bars, ± SD. *** *P* < 0.001, **** *P* < 0.0001, by one-way ANOVA (B and C)

Since KAP1 KD reduced MYCN protein expression in tumors, we hypothesized that KAP1 might regulate MYCN transcription or translation. To evaluate this, we conducted WB analysis and observed a significant decrease in MYCN protein levels upon KAP1 KD (Fig. 3A). Additionally, KAP1 KD resulted in a significant reduction in MYCN mRNA levels (Fig. 3B). Re-expression of KAP1 upregulated MYCN protein and mRNA levels (Fig. S1C and S1D) and promoted colony formation (Fig. S1E and S1F). These results indicate that KAP1 promotes NB progression by up-regulating MYCN expression.

Accumulated data have highlighted the importance of the tight regulation of mRNA stability in the control of gene expression [28, 29]. Thus, we assessed whether KAP1 mediated MYCN mRNA stability. As shown in Fig. 3C, KAP1 KD markedly promoted the degradation of MYCN mRNA. In all, our data indicate that KAP1 regulates MYCN expression by controlling MYCN mRNA stability.

### KAP1 recruits YTHDC1 to increase MYCN mRNA stability

**Fig. 4:**
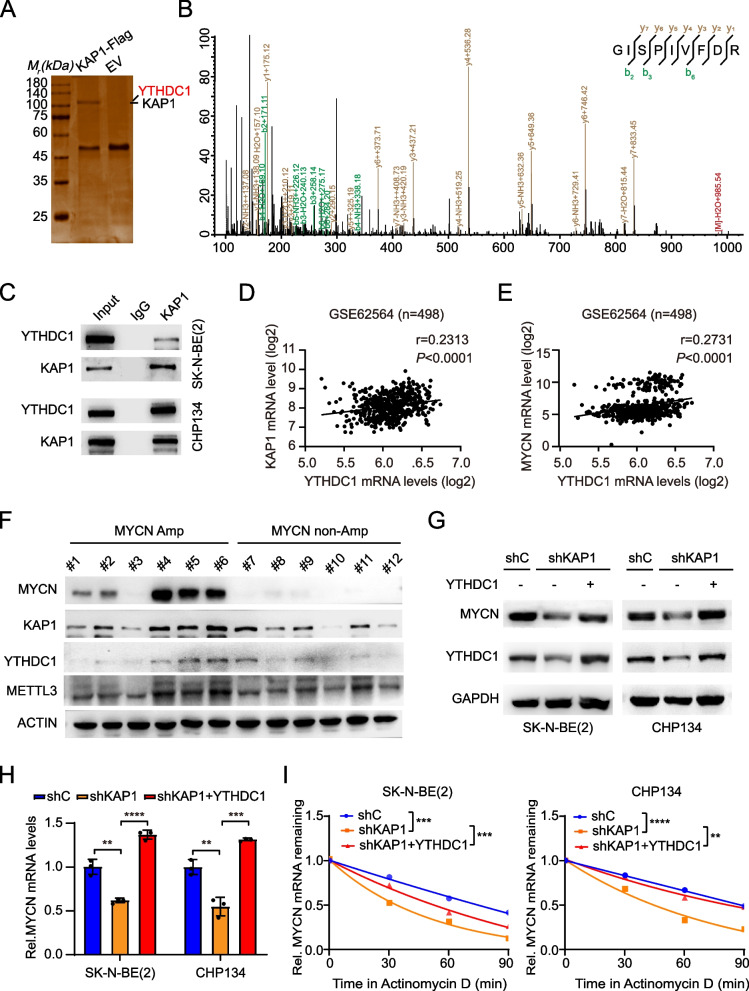
KAP1 recruits YTHDC1 to increase MYCN mRNA stability. **A** and **B**. IP (A) and mass spectrometry analyses (B) of KAP1-associated proteins in SK-N-BE(2) cells. **C** IP analysis of the interaction between KAP1 and YTHDC1 in SK-N-BE(2) and CHP134 cells. **D** Correlation between YTHDC1 and KAP1 in tumors from NB patients. **E** Correlation between YTHDC1 and MYCN in tumors from NB patients. **F** The protein levels of MYCN, KAP1, YTHDC1 and METTL3 in a panel of NB tissue. **G** The protein levels of MYCN and YTHDC1 in SK-N-BE(2) and CHP134 cells expressing shC, shKAP1 and shKAP1 with ectopic YTHDC1. **H** The mRNA levels of MYCN in SK-N-BE(2) and CHP134 cells expressing shC, shKAP1 and shKAP1 with ectopic YTHDC1. **I** The pace of MYCN mRNA degradation in SK-N-BE(2) and CHP134 cells expressing shC, shKAP1 and shKAP1 with ectopic YTHDC1. Data are representative of three independent experiments in H and I. Error bars, ± SD. ** *P* < 0.01, *** *P* < 0.001, **** *P* < 0.0001, by Spearman correlation test (D and E), by unpaired two-tailed *t*-test (H and I)

To reveal the mechanism by which KAP1 regulates the stability of MYCN mRNA, we employed immunoprecipitation (IP) and mass spectrum analysis of KAP1-containing complexes in SK-N-BE(2) cells (Fig. 4A). Mass spectrometry identified a total of 389 unique peptides (Table S3), which were the ionized products of 83 different proteins predicted by proteomic algorithm. Among these putative KAP1-binding proteins, a total of 6 unique peptides of YTHDC1 were identified (Fig. 4B and Table S3). Then, the interaction between KAP1 and YTHDC1 was validated by IP in SK-N-BE(2) and CHP134 cells (Fig. 4C). Besides, there was a positive correlation between the mRNA expression of YTHDC1 and both KAP1 (Fig. 4D) and MYCN (Fig. 4E). Concordantly, YTHDC1 protein levels were positively correlated with KAP1 and MYCN in a panel of clinical NB specimens (Fig. 4F). For YTHDC1 is an RNA m^6^A reader [30, 31], which participates in regulating mRNA stability [32, 33], we focused on YTHDC1 as the cofactor of KAP1 in NB.

To determine the significance of YTHDC1 in KAP1-regulated MYCN mRNA stability, we performed WB and mRNA stability assays. The results revealed that the introduction of YTHDC1 in KAP1 KD cells not only restored the reduced levels of MYCN protein and mRNA expressions, but also improved the stability of MYCN mRNA (Fig. 4G-I). In addition, we found that KAP1 KD reduced YTHDC1 protein levels (Fig. 4G). Consistent with the role of KAP1 in NB, the elevated expression of YTHDC1 in NB was found to be a warning sign (Fig. S2A-S2C). High YTHDC1 levels were significantly associated with worse event-free survival (Fig. S2D and S2E). YTHDC1 KD slowed down NB cell proliferation (Fig. S2F). Taken together, these data indicate that KAP1 interacts with YTHDC1 to regulate the stability of MYCN mRNA and sustain NB tumor malignancy.

### KAP1 protects YTHDC1 protein degradation

**Fig. 5:**
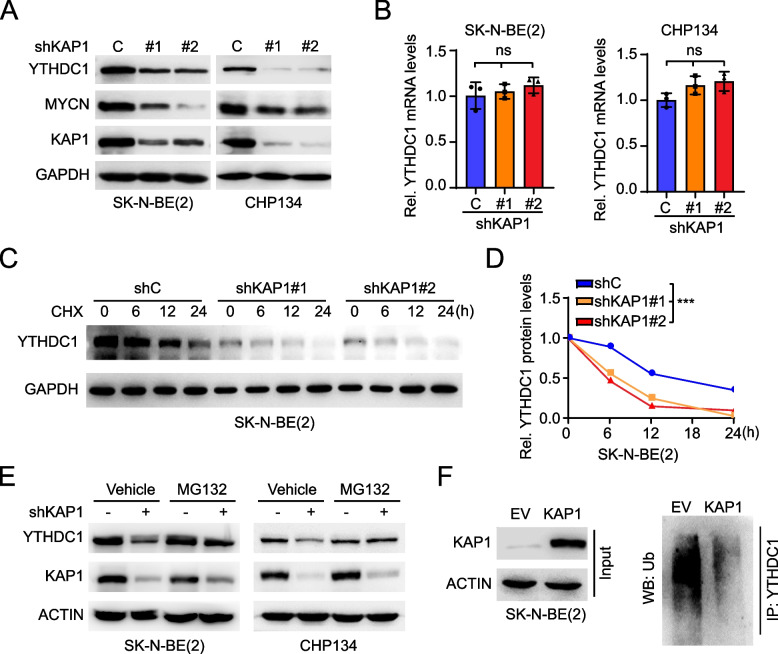
KAP1 protects YTHDC1 protein degradation. **A** Effects of KAP1 knockdown (KD) on the protein levels of YTHDC1 in SK-N-BE(2) and CHP134 cells. **B** Effects of KAP1 KD on the mRNA levels of YTHDC1 in SK-N-BE(2) and CHP134 cells. **C** Effects of KAP1 KD on YTHDC1 protein degradation in SK-N-BE(2) cells. Cells were treated with cyclohexane (CHX) at indicated times. **D** Relative YTHDC1 protein levels recorded in C. **E** Effects of MG132 treatment on YTHDC1 expression in KAP1 KD cells. SK-N-BE(2) and CHP134 cells were pre-treated with or without MG132(10 μM) for 6 h. **F** IP analysis of YTHDC1 ubiquitination in SK-N-BE(2) cells expressing vector or KAP1. Data are representative of three independent experiments in B and D. Error bars, ± SD. ns. not significant, *** *P* < 0.001, by one-way ANOVA (B and D)

Since KAP1 KD reduced YTHDC1 protein expression, we further investigated the relation between KAP1 and YTHDC1. As shown in Fig. 5A and B, KAP1 KD decreased YTHDC1 protein levels but not impaired its mRNA expression. Although accumulated data indicate KAP1 as an E3 ubiquitin ligase to ubiquitin AMPK and p53 [14, 15], it is also shown to protect TRIM24 from SPOP-mediated degradation and promote prostate cancer progression [34]. Thus, we hypothesized that KAP1 protected YTHDC1 protein degradation in NB.

To examine this, we treated KAP1 KD and control cells with cycloheximide, an inhibitor of protein biosynthesis, to monitor YTHDC1 protein degradation. As shown in Fig. 5C and D, KAP1 KD enhanced YTHDC1 degradation in NB cells. Treatment with MG132, a selective inhibitor of proteasome, restored KAP1 KD-reduced YTHDC1 protein expression (Fig. 5E). We further overexpressed KAP1 in SK-N-BE(2) cells and found that ectopic expression of KAP1 decreased YTHDC1 ubiquitination (Fig. 5F). These data demonstrate that KAP1 protects YTHDC1 protein by blocking its ubiquitination and degradation.

### KAP1 interacts with YTHDC1 and METTL3 to sustain MYCN mRNA stability

**Fig. 6:**
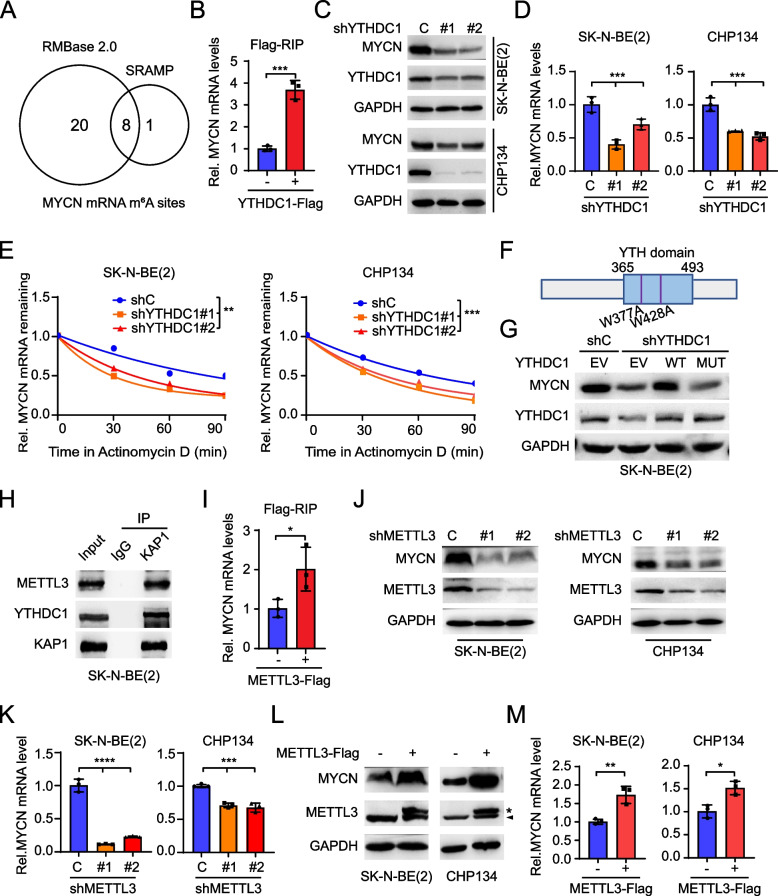
KAP1 enhances MYCN mRNA stability by interacting with YTHDC1 and METTL3. **A** Venn diagram of candidate m^6^A sites of MYCN predicted by both SRAMP and RMBase websites. **B** The interaction between YTHDC1 and MYCN mRNA analyzed by RIP-qPCR assay. **C** Effects of YTHDC1 knockdown (KD) on the protein levels of MYCN in SK-N-BE(2) and CHP134 cells. **D** Effects of YTHDC1 KD on the mRNA levels of MYCN in SK-N-BE(2) and CHP134 cells. **E** Effects of YTHDC1 KD on MYCN mRNA degradation in SK-N-BE(2) and CHP134 cells. Cells were treated with Actinomycin D at indicated times. **F** The diagram of protein domain and mutation sites of YTHDC1. **G** The protein levels of MYCN in YTHDC1-KD cells after being forced expression of wide-type YTHDC1 or YTHDC1^W377A/W428A^. **H** IP analysis of the interaction between KAP1, YTHDC1 and METTL3 in SK-N-BE(2) cells. **I** The interaction between METTL3 and MYCN mRNA analyzed by RIP-qPCR assay. **J** Effects of METTL3 KD on the protein levels of MYCN in SK-N-BE(2) and CHP134 cells. **K** Effects of METTL3 KD on the mRNA levels of MYCN in SK-N-BE(2) and CHP134 cells. **L** Effects of METTL3 overexpression on the protein levels of MYCN in SK-N-BE(2) and CHP134 cells. *, Flag-tagged METTL3. ^▲^, endogenous METTL3. **M** Effects of METTL3 overexpression on the mRNA levels of MYCN in SK-N-BE(2) and CHP134 cells. Data are representative of three independent experiments in B, D, E, I, K and M. Error bars, ± SD. * *P* < 0.05, ** *P* < 0.01, *** *P* < 0.001, **** *P* < 0.0001, by unpaired two-tailed *t*-test (B, I and M), by one-way ANOVA (D, E and K)

The modification of MYCN with m^6^A plays a crucial role in regulating MYCN mRNA stability [35, 36]. Through a comprehensive search of the SRAMP and RMBase 2.0 databases, we identified 8 potential m^6^A modification sites within the MYCN mRNA that were consistently predicted by both platforms (Fig. 6A). Subsequently, RIP-qPCR was conducted using an anti-Flag antibody in SK-N-BE(2)/YTHDC1-Flag cells, confirming the specific interaction between YTHDC1 and MYCN mRNA (Fig. 6B). YTHDC1 KD in SK-N-BE(2) and CHP134 cells not only led a significant reduction in MYCN mRNA and protein levels (Fig. 6C and D), but also a profound impairment of MYCN mRNA stability (Fig. 6E). Consistently, ectopic expression of YTHDC1 increased MYCN protein and mRNA expression (Fig. S3A and S3B). Additionally, we generated a two-point mutant of YTHDC1, W377A and W428A, which demonstrated an inhibitory effect on YTHDC1 binding to m^6^A (Fig. 6F) [31]. Re-expression of YTHDC1 WT but not the YTHDC1^W377A/W428A^ MUT restored MYCN protein expression reduced by YTHDC1 KD (Fig. 6G), indicating that KAP1 safeguards YTHDC1 from protein degradation and modulates MYCN mRNA stability in an m^6^A-dependent manner.

METTL3 is the sole catalytic subunit of m^6^A methyltransferase complex [37]. In mouse embryonic stem cells, METTL3 interacts with KAP1 and YTHDC1 to regulate heterochromatin [23]. Here, we performed IP and found that KAP1 formed a complex with METTL3 and YTHDC1 in NB cells (Fig. 6H). The expression levels of KAP1, YTHDC1, and METTL3 were relatively elevated in MYCN amplified cell lines, SK-N-BE(2) and CHP134 (Fig. S3C and S3D). RIP-qPCR confirmed the binding of METTL3 to MYCN mRNA (Fig. 6I). Although the heightened expression of METTL3 did not demonstrate a significant correlation with patients’ survival, tumors from high-risk group patients exhibited elevated levels of METTL3 (Fig. S3E and S3F). METTL3 KD decreased MYCN protein expression and mRNA levels (Fig. 6J and K). Consistently, ectopic expression of METTL3 increased MYCN protein expression and mRNA levels (Fig. 6L and M). Taken together, our data demonstrate that KAP1 forms a complex with YTHDC1 and METTL3 to sustain m^6^A-modified MYCN mRNA stability.

### METTL3 inhibitor and chemotherapy synergize to reduce MYCN-amplified neuroblastoma tumorigenicity

**Fig. 7:**
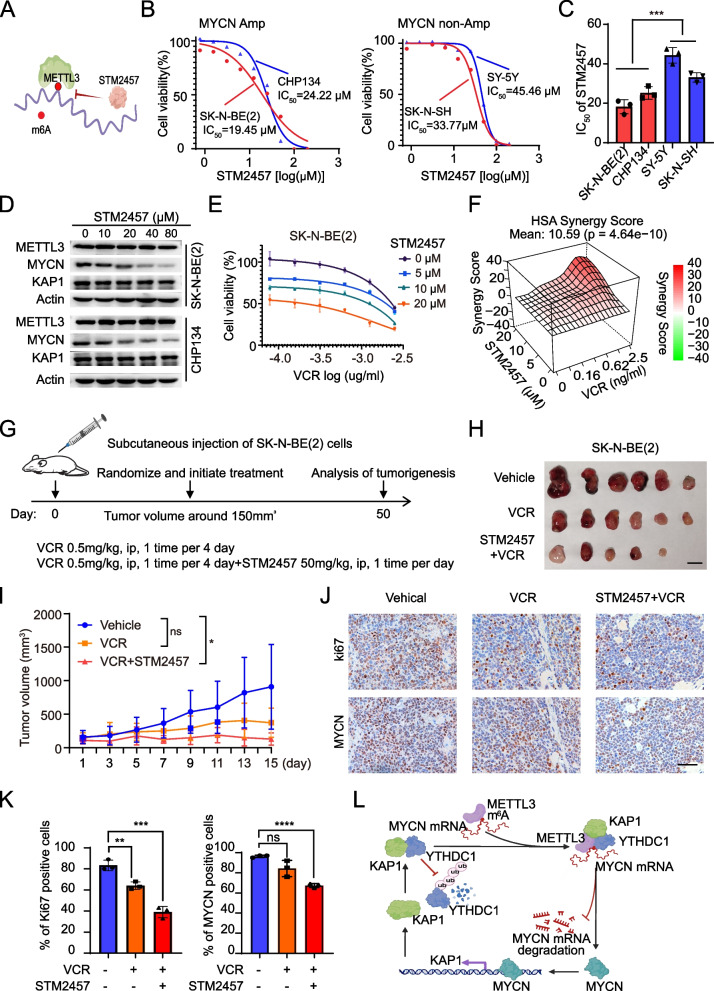
Reducing N6-Methyladenosine levels of MYCN mRNA decreases neuroblastoma growth. **A** The working model of STM2457-inhibited m^6^A modification. **B** Dose-response curves of SK-N-BE(2), CHP134, SY-5Y and SK-N-SH cells to STM2457. **C** Quantification of half-maximal inhibitory concentrations (IC_50_) in B. **D** Effects of STM2457 on the protein levels of MYCN, KAP1 and METTL3 in SK-N-BE(2) and CHP134 cells. **E** Cell viability curves of SK-N-BE(2) cells treated with a range of VCR (0–2.5 ng/ml) in combination with 0, 5, 10, or 20 μM STM2457. **F** The open-source R package SynergyFinder was used to calculate HSA synergy scores and plot the dose–response curve for the treatment of SK-N-BE(2) cells with STM2457 and VCR in E. The resulting HSA synergy scores were depicted on the z-axis of the HSA graph and served as indicators for identifying the concentrations at which synergy was observed. **G** Experimental setup for tumor-bearing mice treated with indicated reagents. **H** Images of subcutaneous xenograft tumors at the endpoints treated with indicated reagents. Scale bars: 1 cm. **I** Tumor growth curves recorded in the xenograft mice treated with indicated reagents. **J** Representative images of IHC staining of MYCN and Ki-67 in the xenograft mice. Scale bars: 75 μm. **K** Quantification of MYCN and Ki67 positive rates in J. **L** Model of KAP1-mediated tumorigenic effect in MYCN-amplified NB. Data are representative of three independent experiments in B, E and K. Error bars, ± SD. ns, not significant. * *P* < 0.05, ** *P* < 0.01, *** *P* < 0.001, **** *P* < 0.0001, by unpaired two-tailed *t*-test (C, I, and K)

STM2457 is a novel and selective inhibitor of m^6^A modification, which has shown therapeutic effects in acute myeloid leukemia (AML) [38] and hepatocellular carcinoma [39], but its antineoplastic effect in NB is not definite. We performed cell survival analysis and found that STM2457 reduced the cell viability of NB cells in a dose-dependent manner (Fig. 7B). Its half-maximal inhibitory concentrations (IC_50_) in MYCN-amplified SK-N-BE(2) and CHP134 cells were lower than that in non-MYCN amplified SK-N-SH and SY-5Y cells (Fig. 7C). STM2457 treatment also decreased MYCN, but not KAP1, protein levels (Fig. 7D). These results support the potential of STM2457 for NB treatment and its specific responsiveness to MYCN amplification.

Vincristine (VCR) is one of the most common chemotherapy medicines in patients with relapsed/refractory NB [40]. Although intensive chemotherapy has greatly improved the survival rate, it is often accompanied by severe and lifelong side effects. There is an urgent need to develop more selective and less toxic chemotherapy for NB. Combined treatment of SK-N-BE(2) cells by STM2457 and VCR resulted in a strong synergistic anti-proliferative effect across a range of concentrations (Fig. 7E and F), suggesting a new route for NB treatment.

To further determine whether the combination of STM2457 and VCR represented a more effective and safer therapeutic schedule for MYCN-amplified NB, we assessed its effect on tumor growth in vivo (Fig. 7G). We found that the growth of tumors treated with STM2457 and VCR was significantly slower than that in prescribing VCR alone and vehicle control (Fig. 7H and I). Related drugs have no overt toxicity or effect on mouse body weight, temperature, appearance or activity. Consistent with our previous in vitro findings, we also observed a marked decrease in MYCN and Ki-67 proteins in the tumors of the combination group (Fig. 6J and K), Taken together, these results suggested that m^6^A modification is a potential therapeutic target for NB. Administration of STM2457 in combination with VCR synergistically enhances cell-killing efficacy.

## Discussion

In this research, we present a novel potential treatment approach for MYCN-amplified NB by focusing on KAP1 to decrease the stability of MYCN mRNA. KAP1 exhibits high expression levels in MYCN-amplified specimens and serves as an independent prognostic indicator for NB. KAP1 forms a complex with YTHDC1 and METTL3 to regulate the stability of m^6^A-modified MYCN mRNA. Reducing KAP1 expression results in decreased stability of YTHDC1 protein, causing degradation of MYCN mRNA. The combination of a METTL3-targeted inhibitor with chemotherapy yields a synergistic effect in inhibiting NB proliferation by impeding the m^6^A modification of MYCN mRNA (Fig. 7L).

Our findings indicate that KAP1 serves as a novel prognostic marker for NB. KAP1 has been demonstrated to function in the regulation of gene expression [41], DNA damage [42, 43], protein ubiquitination [44], epithelial to mesenchymal transition [45], stem cell pluripotency [46], autophagy [47], and genome stability [19, 20]. To date, however, its relevance in cancer remains elusive. Higher KAP1 levels are related to more malignant cervical cancer [48]. The upregulation of KAP1 in lung cancer is correlated with increasing invasive features [44]. Additionally, KAP1 serves as a prognostic biomarker for tumor classification, aiding in the differentiation of high-grade bladder cancer [49]. On the contrary, KAP1 is also proven to be a tumor suppressor in melanoma [50]. In early-stage lung cancer, KAP1 high expression is associated with improved overall survival [51]. Liver-specific ablation of KAP1 in mice leads to an increase in male-predominant hepatic adenoma [52]. Here, our data strongly indicate that KAP1 is an oncogene in NB. High KAP1 expression is related to more rapid tumor proliferation. NB patients with high KAP1 expression have poor survival. Moreover, KAP1 plays a crucial role in preserving MYCN mRNA stability in MYCN-amplified NB.

Our findings demonstrate that KAP1 and MYCN establish a positive feedback loop. In clinical samples, elevated KAP1 expression is observed in MYCN-amplified tumors, demonstrating co-expression of KAP1 and MYCN at both mRNA and protein levels. Amplification of MYCN enhances KAP1 transcription, while elevated levels of KAP1, in turn, increase the stability of MYCN mRNA. This feedback loop significantly amplifies the tumorigenic potential of NB, underscoring the pivotal role of KAP1 in the context of MYCN-amplified NB.

We further indicate that KAP1 protects YTHDC1 degradation. YTHDC1, an m^6^A reader [53], is detected to be highly expressed in various solid cancers, including breast cancer [54], esophageal cancer [55] and hepatocellular carcinoma [56]. When YTHDC1 is overexpressed in triple-negative breast cancer, there is a notable increase in lung metastases in vivo [54]. Here, we demonstrate that upregulated KAP1 by MYCN interacts with YTHDC1 and protects YTHDC1 from ubiquitination and degradation, leading to enhanced tumor cell proliferation.

We demonstrate that KAP1 forms a complex with YTHDC1 and METTL3 to enhance the stability of the m^6^A-modified MYCN mRNA. As the sole nuclear RNA m^6^A reader in the YTH domain family, YTHDC1 plays unique roles in regulating nuclear RNA alternative polyadenylation, splicing, translation, nuclear export, and stability in an m^6^A-dependent manner [57, 58]. m^6^A modification is the most prevalent, abundant and conserved internal modification of RNA in eukaryotic cells [59]. In vivo, m^6^A modification is dynamically installed by m^6^A methyltransferase (such as METTL3/14, WTAP), removed by m^6^A demethylase (such as FTO, ALKBH5), and recognized by m^6^A reading molecules (such as YTHDC1/2, YTHDF1/2/3) [53, 60]. Accumulating studies suggest that m^6^A methylation governs the fate of modified RNA and participates in cancer initiation, development, and maintenance [61–63]. However, the precise role of m^6^A modification on MYCN RNA and NB remains unknown. Here, our data reveal that catalyzing and decoding m^6^A modification on MYCN is essential to maintain its expression. KAP1 forms a complex with YTHDC1 and METTL3 to increase the stability of m^6^A-modified MYCN mRNA. Inhibiting either METTL3 or YTHDC1 could decrease MYCN expression.

Moreover, our research shows that when we use a METTL3-targeted inhibitor to block m^6^A modification of MYCN mRNA, it combines effectively with VCR to inhibit the growth of NB cells. STM2457 is a small-molecule inhibitor of METTL3, which could selectively reduce m^6^A levels on target mRNA and regulate their expression levels [38]. STM2457 demonstrates cytotoxicity, exhibiting a specific inhibition of primary tumor cells with an IC_50_ of 38.35 μM in SHH subgroup medulloblastoma [64]. Additionally, STM2457 leads to a reduction in the growth of normal cerebellar neurons, albeit with a higher IC_50_ of 80.54 μM [64]. Here, we observe that STM2457 displays toxicity towards all tested NB cell lines, but cells with MYCN amplification show a heightened sensitivity to STM2457 compared to those without MYCN amplification. Furthermore, the combination therapy involving STM2457 and VCR shows enhanced efficacy in preventing tumor progression and suppressing MYCN expressions. It has the potential to be an effective therapeutic method against MYCN-amplified NB.

## Conclusions

Taken together, our data demonstrate that KAP1 forms a complex with YTHDC1 and METTL3, which protects YTHDC1 protein degradation and maintains m^6^A-modified MYCN mRNA stability. The destabilization of MYCN mRNA by targeting KAP1 and inhibiting METTL3 activity represents an alternative therapeutic strategy for MYCN-amplified NB. While more studies are necessary, this new therapeutic strategy might bring bright prospects in the treatment of MYCN-amplified NB.

### Supplementary Information

**Supplementary Material 1.**

## Data Availability

All data needed to evaluate the conclusions in the paper are present in the paper and/or the Supplementary Materials. Additional data related to this paper may be requested from the authors. RNA-seq data are available in Gene Expression Omnibus (GEO) with accession code GSE62564.

## Abbreviations

NB: Neuroblastoma
m^6^A: N6-methyladenosine
TRIM: Tripartite-motif
ATCC: American type culture collection
ECACC: European collection of authenticated cell cultures
DMEM: Dulbecco’s modified eagle’s medium
FBS: Fetal bovine serum
RPMI: Roswell park memorial institute
IRB: Institutional review board
WB: Western blotting
IHC: Immunohistochemistry
H&E: Hematoxylin&eosin
Co-IP: Co-immunoprecipitation
RIP: RNA immunoprecipitation
GEO: Gene expression omnibus
TSS: Transcription start sites
AML: Acute myeloid leukemia
IC_50_: Half-maximal inhibitory concentrations
VCR: Vincristine

## References

[1] Swift CC, Eklund MJ, Kraveka JM, Alazraki AL (2018). Updates in diagnosis, management, and treatment of neuroblastoma. Radiographics..

[2] Schmitt-Hoffner F, Van Rijn S, Toprak UH, Mauermann M, Rosemann F, Heit-Mondrzyk A (2021). FOXR2 stabilizes MYCN protein and identifies non-MYCN-amplified neuroblastoma patients with unfavorable outcome. J Clin Oncol.

[3] Otte J, Dyberg C, Pepich A, Johnsen JI (2020). MYCN function in neuroblastoma development. Front Oncol.

[4] Pinto NR, Applebaum MA, Volchenboum SL, Matthay KK, London WB, Ambros PF (2015). Advances in risk classification and treatment strategies for neuroblastoma. J Clin Oncol.

[5] Fletcher JI, Ziegler DS, Trahair TN, Marshall GM, Haber M, Norris MD (2018). Too many targets, not enough patients: rethinking neuroblastoma clinical trials. Nat Rev Cancer.

[6] Wolpaw AJ, Bayliss R, Büchel G, Dang CV, Eilers M, Gustafson WC (2021). Drugging the "Undruggable" MYCN oncogenic transcription factor: overcoming previous obstacles to impact childhood cancers. Cancer Res.

[7] Otto T, Horn S, Brockmann M, Eilers U, Schüttrumpf L, Popov N (2009). Stabilization of N-Myc is a critical function of Aurora a in human neuroblastoma. Cancer Cell.

[8] Gustafson WC, Meyerowitz JG, Nekritz EA, Chen J, Benes C, Charron E (2014). Drugging MYCN through an allosteric transition in Aurora kinase a. Cancer Cell.

[9] Puissant A, Frumm SM, Alexe G, Bassil CF, Qi J, Chanthery YH (2013). Targeting MYCN in neuroblastoma by BET bromodomain inhibition. Cancer Discov.

[10] Rohaan MW, Gomez-Eerland R, Van Den Berg JH, Geukes Foppen MH, Van Zon M, Raud B (2022). MART-1 TCR gene-modified peripheral blood T cells for the treatment of metastatic melanoma: a phase I/IIa clinical trial. Immunooncol Technol.

[11] Toyoshima M, Howie HL, Imakura M, Walsh RM, Annis JE, Chang AN (2012). Functional genomics identifies therapeutic targets for MYC-driven cancer. Proc Natl Acad Sci USA.

[12] Czerwińska P, Mazurek S, Wiznerowicz M (2017). The complexity of TRIM28 contribution to cancer. J Biomed Sci.

[13] Huang N, Sun X, Li P, Liu X, Zhang X, Chen Q (2022). TRIM family contribute to tumorigenesis, cancer development, and drug resistance. Exp Hematol Oncol.

[14] Pineda CT, Ramanathan S, Fon Tacer K, Weon JL, Potts MB, Ou YH (2015). Degradation of AMPK by a cancer-specific ubiquitin ligase. Cell..

[15] Wang C, Ivanov A, Chen L, Fredericks WJ, Seto E, Rauscher FJ (2005). MDM2 interaction with nuclear corepressor KAP1 contributes to p53 inactivation. EMBO J.

[16] Ma X, Yang T, Luo Y, Wu L, Jiang Y, Song Z (2019). TRIM28 promotes HIV-1 latency by SUMOylating CDK9 and inhibiting P-TEFb. Elife..

[17] Li M, Xu X, Chang CW, Liu Y (2020). TRIM28 functions as the SUMO E3 ligase for PCNA in prevention of transcription induced DNA breaks. Proc Natl Acad Sci USA.

[18] Diao Z, Ji Q, Wu Z, Zhang W, Cai Y, Wang Z (2021). SIRT3 consolidates heterochromatin and counteracts senescence. Nucleic Acids Res.

[19] Rowe HM, Jakobsson J, Mesnard D, Rougemont J, Reynard S, Aktas T (2010). KAP1 controls endogenous retroviruses in embryonic stem cells. Nature..

[20] Ecco G, Cassano M, Kauzlaric A, Duc J, Coluccio A, Offner S (2016). Transposable elements and their KRAB-ZFP controllers regulate gene expression in adult tissues. Dev Cell.

[21] Bacon CW, Challa A, Hyder U, Shukla A, Borkar AN, Bayo J (2020). KAP1 is a chromatin reader that couples steps of RNA polymerase II transcription to sustain oncogenic programs. Mol Cell.

[22] Mcnamara RP, Reeder JE, Mcmillan EA, Bacon CW, Mccann JL, D'orso I (2016). KAP1 recruitment of the 7SK snRNP complex to promoters enables transcription elongation by RNA polymerase II. Mol Cell.

[23] Xu W, Li J, He C, Wen J, Ma H, Rong B (2021). METTL3 regulates heterochromatin in mouse embryonic stem cells. Nature..

[24] Percharde M, Lin CJ, Yin Y, Guan J, Peixoto GA, Bulut-Karslioglu A (2018). A LINE1-Nucleolin partnership regulates early development and ESC identity. Cell..

[25] Sang Y, Li Y, Song L, Alvarez AA, Zhang W, Lv D (2018). TRIM59 promotes Gliomagenesis by inhibiting TC45 Dephosphorylation of STAT3. Cancer Res.

[26] Song L, Yu B, Yang Y, Liang J, Zhang Y, Ding L (2021). Identification of functional cooperative mutations of GNAO1 in human acute lymphoblastic leukemia. Blood..

[27] Yang Y, Wang S, Cai J, Liang J, Zhang Y, Xie Y (2023). Targeting ARHGEF12 promotes neuroblastoma differentiation, MYCN degradation, and reduces tumorigenicity. Cell Oncol (Dordr).

[28] Ghosh S, Jacobson A (2010). RNA decay modulates gene expression and controls its fidelity. Wiley Interdiscip Rev RNA.

[29] Boo SH, Kim YK (2020). The emerging role of RNA modifications in the regulation of mRNA stability. Exp Mol Med.

[30] Zhang Z, Theler D, Kaminska KH, Hiller M, De La Grange P, Pudimat R (2010). The YTH domain is a novel RNA binding domain. J Biol Chem.

[31] Xu C, Wang X, Liu K, Roundtree IA, Tempel W, Li Y (2014). Structural basis for selective binding of m6A RNA by the YTHDC1 YTH domain. Nat Chem Biol.

[32] Li F, Yi Y, Miao Y, Long W, Long T, Chen S (2019). N(6)-Methyladenosine modulates nonsense-mediated mRNA decay in human glioblastoma. Cancer Res.

[33] Liu S, Li G, Li Q, Zhang Q, Zhuo L, Chen X (2020). The roles and mechanisms of YTH domain-containing proteins in cancer development and progression. Am J Cancer Res.

[34] Fong KW, Zhao JC, Song B, Zheng B, Yu J (2018). TRIM28 protects TRIM24 from SPOP-mediated degradation and promotes prostate cancer progression. Nat Commun.

[35] Zhu K, Gao T, Wang Z, Zhang L, Tan K, Lv Z (2023). RNA N6-methyladenosine reader IGF2BP3 interacts with MYCN and facilitates neuroblastoma cell proliferation. Cell Death Discov..

[36] Cheng J, Xu L, Deng L, Xue L, Meng Q, Wei F (2020). RNA N(6)-methyladenosine modification is required for miR-98/MYCN axis-mediated inhibition of neuroblastoma progression. Sci Rep.

[37] Zeng C, Huang W, Li Y, Weng H (2020). Roles of METTL3 in cancer: mechanisms and therapeutic targeting. J Hematol Oncol.

[38] Yankova E, Blackaby W, Albertella M, Rak J, De Braekeleer E, Tsagkogeorga G (2021). Small-molecule inhibition of METTL3 as a strategy against myeloid leukaemia. Nature..

[39] Wang L, Yang Q, Zhou Q, Fang F, Lei K, Liu Z (2023). METTL3-m(6)A-EGFR-axis drives lenvatinib resistance in hepatocellular carcinoma. Cancer Lett.

[40] Zhu J, Wang J, Sun F, Zhen Z, Chen T, Lu S (2022). Vincristine, Irinotecan, and Temozolomide in patients with relapsed/refractory neuroblastoma. Front Oncol.

[41] Bunch H, Zheng X, Burkholder A, Dillon ST, Motola S, Birrane G (2014). TRIM28 regulates RNA polymerase II promoter-proximal pausing and pause release. Nat Struct Mol Biol.

[42] Noon AT, Shibata A, Rief N, Löbrich M, Stewart GS, Jeggo PA (2010). 53BP1-dependent robust localized KAP-1 phosphorylation is essential for heterochromatic DNA double-strand break repair. Nat Cell Biol.

[43] White D, Rafalska-Metcalf IU, Ivanov AV, Corsinotti A, Peng H, Lee SC (2012). The ATM substrate KAP1 controls DNA repair in heterochromatin: regulation by HP1 proteins and serine 473/824 phosphorylation. Mol Cancer Res.

[44] Jin JO, Lee GD, Nam SH, Lee TH, Kang DH, Yun JK (2021). Sequential ubiquitination of p53 by TRIM28, RLIM, and MDM2 in lung tumorigenesis. Cell Death Differ.

[45] Yu C, Zhan L, Jiang J, Pan Y, Zhang H, Li X (2014). KAP-1 is overexpressed and correlates with increased metastatic ability and tumorigenicity in pancreatic cancer. Med Oncol.

[46] Li J, Xi Y, Li W, Mccarthy RL, Stratton SA, Zou W (2017). TRIM28 interacts with EZH2 and SWI/SNF to activate genes that promote mammosphere formation. Oncogene..

[47] Song T, Lv S, Ma X, Zhao X, Fan L, Zou Q (2023). TRIM28 represses renal cell carcinoma cell proliferation by inhibiting TFE3/KDM6A-regulated autophagy. J Biol Chem.

[48] Lin LF, Li CF, Wang WJ, Yang WM, Wang DD, Chang WC (2013). Loss of ZBRK1 contributes to the increase of KAP1 and promotes KAP1-mediated metastasis and invasion in cervical cancer. PLoS One.

[49] Agarwal N, Rinaldetti S, Cheikh BB, Zhou Q, Hass EP, Jones RT (2021). TRIM28 is a transcriptional activator of the mutant TERT promoter in human bladder cancer. Proc Natl Acad Sci USA.

[50] Lionnard L, Duc P, Brennan MS, Kueh AJ, Pal M, Guardia F (2019). TRIM17 and TRIM28 antagonistically regulate the ubiquitination and anti-apoptotic activity of BCL2A1. Cell Death Differ.

[51] Chen L, Chen DT, Kurtyka C, Rawal B, Fulp WJ, Haura EB (2012). Tripartite motif containing 28 (Trim28) can regulate cell proliferation by bridging HDAC1/E2F interactions. J Biol Chem.

[52] Bojkowska K, Aloisio F, Cassano M, Kapopoulou A, Santoni De Sio F, Zangger N (2012). Liver-specific ablation of Krüppel-associated box-associated protein 1 in mice leads to male-predominant hepatosteatosis and development of liver adenoma. Hepatology..

[53] Shi H, Wei J, He C (2019). Where, when, and how: context-dependent functions of RNA methylation writers, readers, and erasers. Mol Cell.

[54] Tan B, Zhou K, Liu W, Prince E, Qing Y, Li Y (2022). RNA N(6) -methyladenosine reader YTHDC1 is essential for TGF-beta-mediated metastasis of triple negative breast cancer. Theranostics..

[55] Zhao H, Xu Y, Xie Y, Zhang L, Gao M, Li S (2021). m6A regulators is differently expressed and correlated with immune response of esophageal Cancer. Front Cell Dev Biol.

[56] Liu J, Sun G, Pan S, Qin M, Ouyang R, Li Z (2020). The Cancer genome atlas (TCGA) based m(6) a methylation-related genes predict prognosis in hepatocellular carcinoma. Bioengineered..

[57] Widagdo J, Anggono V, Wong JJ (2022). The multifaceted effects of YTHDC1-mediated nuclear m(6)a recognition. Trends Genet.

[58] Yan H, Zhang L, Cui X, Zheng S, Li R (2022). Roles and mechanisms of the m(6)a reader YTHDC1 in biological processes and diseases. Cell Death Discov.

[59] Jiang X, Liu B, Nie Z, Duan L, Xiong Q, Jin Z (2021). The role of m6A modification in the biological functions and diseases. Signal Transduct Target Ther.

[60] Zaccara S, Ries RJ, Jaffrey SR (2019). Reading, writing and erasing mRNA methylation. Nat Rev Mol Cell Biol.

[61] Sun T, Wu R, Ming L (2019). The role of m6A RNA methylation in cancer. Biomed Pharmacother.

[62] He L, Li H, Wu A, Peng Y, Shu G, Yin G (2019). Functions of N6-methyladenosine and its role in cancer. Mol Cancer.

[63] Liu ZX, Li LM, Sun HL, Liu SM (2018). Link between m6A modification and cancers. Front Bioeng Biotechnol.

[64] Zhang ZW, Teng X, Zhao F, Ma C, Zhang J, Xiao LF (2022). METTL3 regulates m(6)a methylation of PTCH1 and GLI2 in sonic hedgehog signaling to promote tumor progression in SHH-medulloblastoma. Cell Rep.

