# Supplementary material for: KAP1 stabilizes MYCN mRNA and promotes neuroblastoma tumorigenicity by protecting the RNA m6A reader YTHDC1 protein degradation
Source: J Exp Clin Cancer Res. 2024 May 14;43:141. doi: 10.1186/s13046-024-03040-9 (PMC11092262; doi:10.1186/s13046-024-03040-9)
Supplement: Supplementary file 1 — Supplementary Material 1. [file 13046_2024_3040_MOESM1_ESM.docx]

Supporting Information

**KAP1 stabilizes MYCN mRNA by protecting the RNA m^6^A reader YTHDC1 protein degradation**

Yi Yang, Yingwen Zhang, Guoyu Chen, Bowen Sun, Fei Luo, Yijin Gao, Haizhong Feng*, and Yanxin Li*.

*Corresponding author: Yanxin Li, E-mail: liyanxin@scmc.com.cn or Haizhong Feng, E-mail: fenghaizhong@sjtu.edu.cn

**
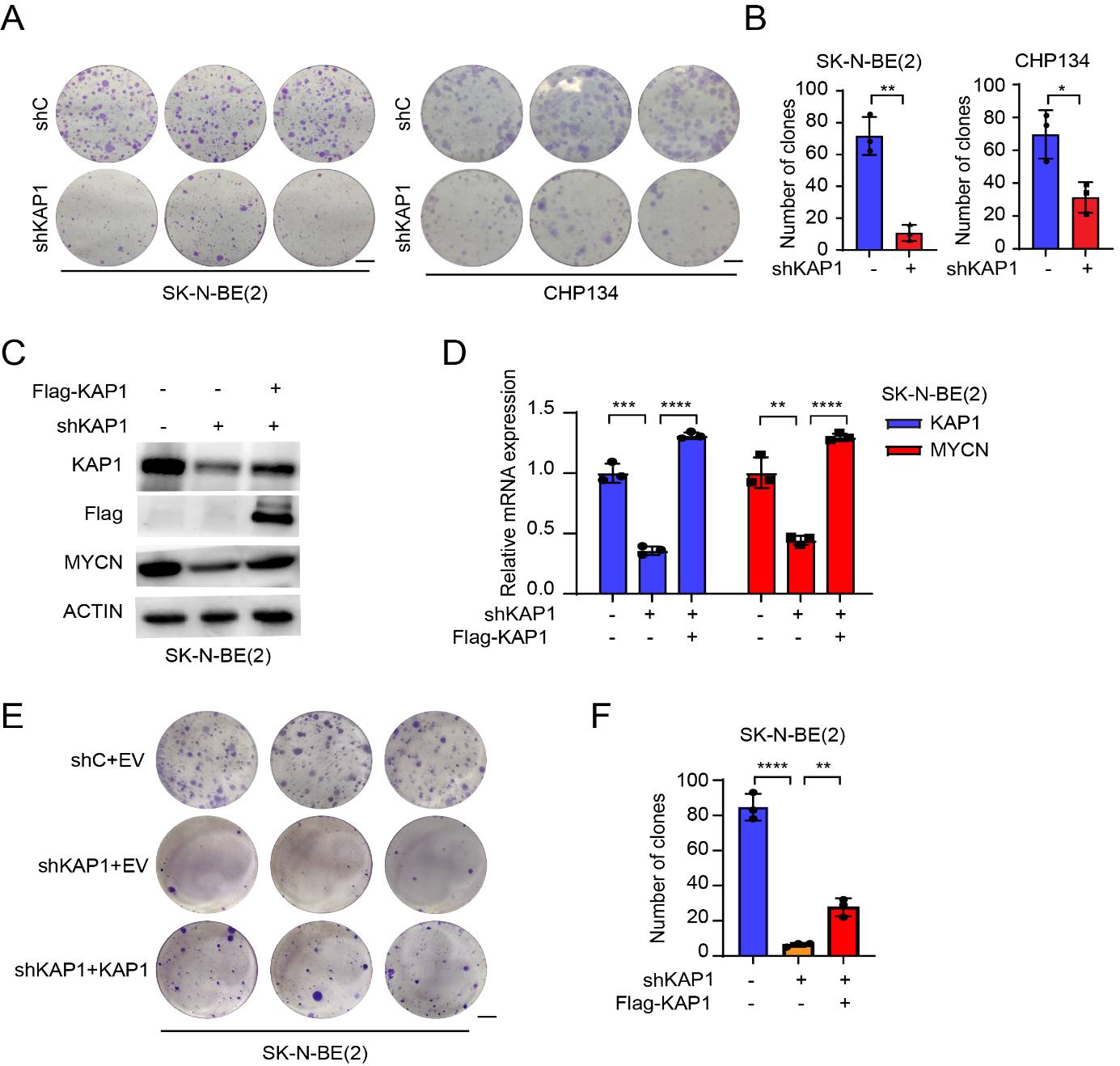
**

**Figure. S1** Re-expression of KAP1 promotes colony formation by up-regulating MYCN expression

**A** Representative images of the colony formation in SK-N-BE(2) and CHP134 cells with modified KAP1 expression. Scale bars: 300 μm. **B** Quantification of colony numbers in A. **C** Effects of re-expression of KAP1 on MYCN protein expression. KAP1 shRNA-resistant Flag-KAP1 was re-expressed in SK-N-BE(2)/shKAP1 cells. **D** Effects of re-expression of KAP1 on MYCN mRNA expression. **E** Representative images of the colony formation of SK-N-BE(2) cells expressing shC, shKAP1 and shKAP1 with ectopic KAP1. Scale bars: 300 μm. **F** Quantification of colony numbers in E. Data are representative of three independent experiments in A and E. Error bars, ± SD. * *P* < 0.05, ** *P* < 0.01, *** *P* < 0.001, **** *P* < 0.0001, by unpaired two-tailed *t*-test (B, D and F).


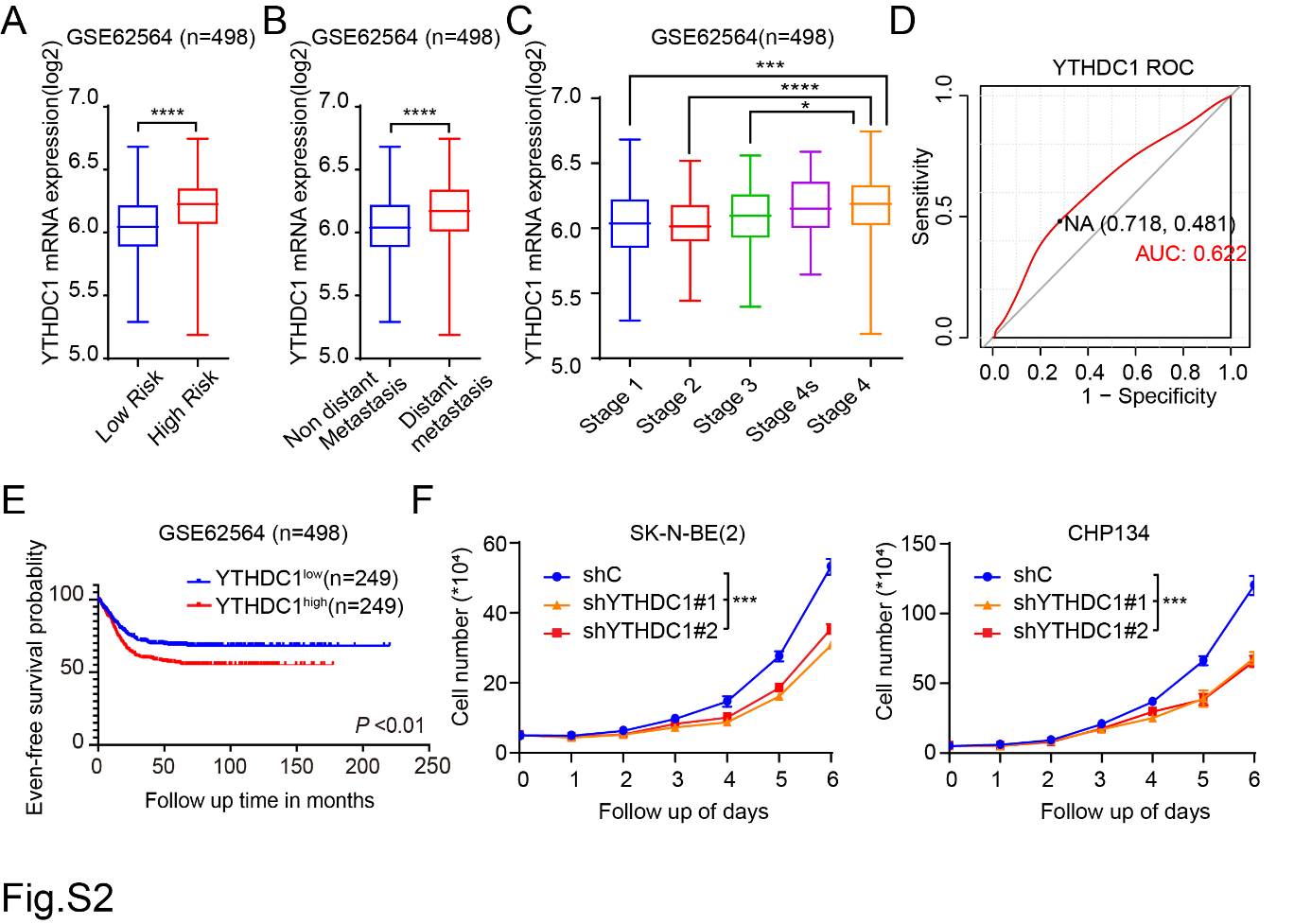


**Figure. S2** YTHDC1 is a prognostic factor in neuroblastoma

**A** The relative expression of YTHDC1 in cohorts grouped by risk stratification. **B** The relative expression of YTHDC1 in cohorts grouped by distant metastasis or not. **C** The relative expression of YTHDC1 in cohorts grouped by INSS stages. **D** ROC curve assessment of the prognostic ability of YTHDC1 expression in NB. **E** Kaplan-Meier survival analysis of NB patients with different levels of YTHDC1 expression. **F** Effects of YTHDC1 knockdown (KD) on cell proliferation in SK-N-BE(2) and CHP134 cells. Data are representative of three independent experiments in F. Error bars, ± SD. * *P* < 0.05, *** *P* < 0.001, **** *P* < 0.0001, by unpaired two-tailed *t*-test (A, B, and C), by one-way ANOVA (F), by log-rank test (E).

**
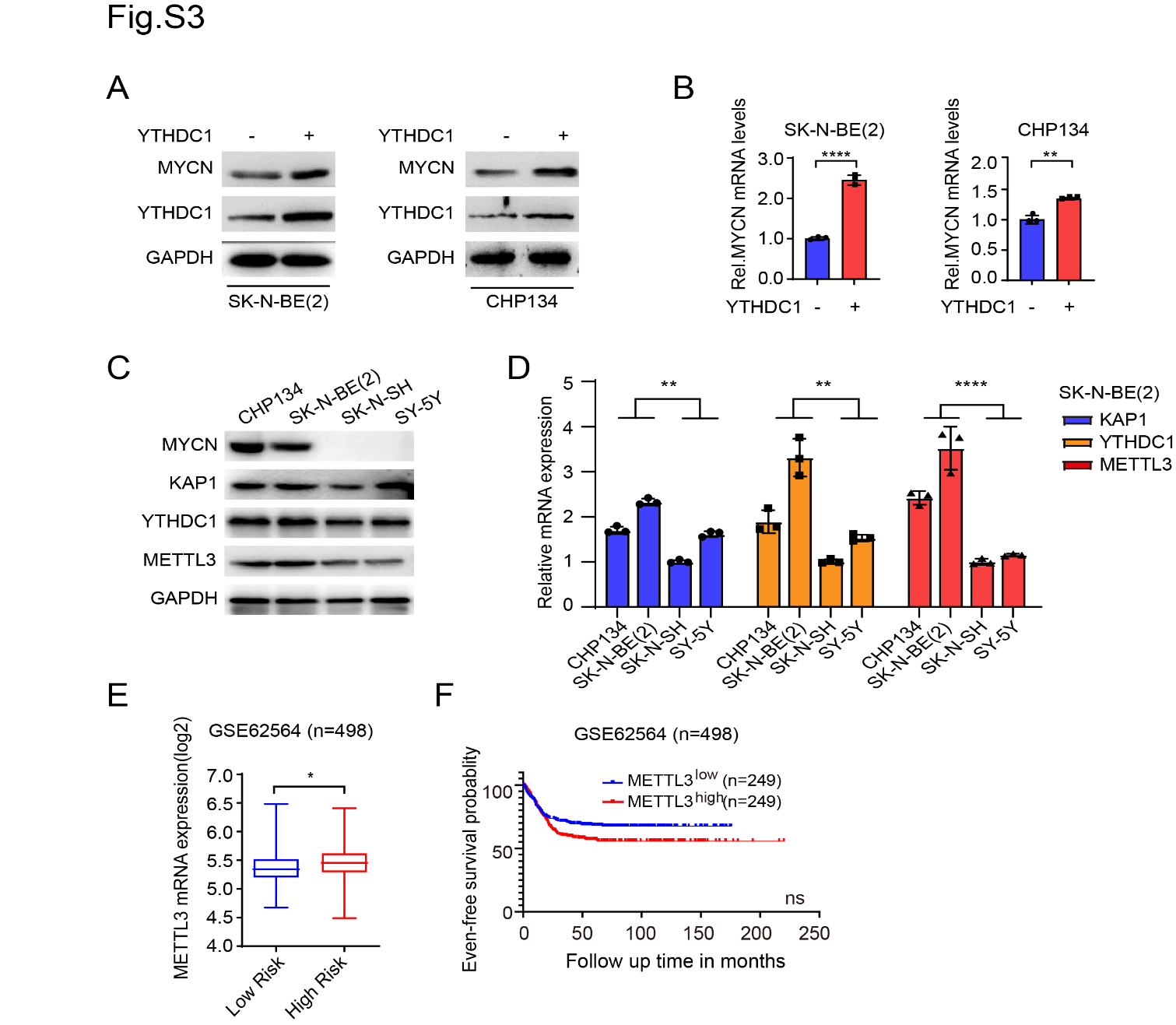
**

**Delete**

**Figure. S3** Ectopic expression of YTHDC1 upregulates MYCN

**A** Effects of YTHDC1 overexpression on the protein levels of MYCN in SK-N-BE(2) and CHP134 cells. **B** Effects of YTHDC1 overexpression on the mRNA levels of MYCN in SK-N-BE(2) and CHP134 cells. **C**. Comparison of protein levels of MYCN, KAP1, YTHDC1, and METTL3 in NB cell lines. **D**. Comparison of mRNA levels of KAP1, YTHDC1, and METTL3 in NB cell lines. **E** The relative expression of METTL3 in cohorts grouped by risk stratification. **F** Kaplan-Meier survival analysis of NB patients with different levels of METTL3 expression. Data are representative of three independent experiments in B and D. Error bars, ± SD. * *P* < 0.05, ** *P* < 0.01, **** *P* < 0.0001, by unpaired two-tailed *t*-test (B, D and E), by log-rank test (F).

**Table S1** shRNA and sgRNA oligos

shKAP1#1 CCTGGCTCTGTTCTCTGTCCT

shKAP1#2 CTGAGACCAAACCTGTGCTTA

shYTHDC1#1 TGCCTCCAGAGAACCTTATAA

shYTHDC1#2 GTCGACCAGAAGATTATGATA

shMETTL3#1 GCCTTAACATTGCCCACTGAT

shMETTL3#2 GCCAAGGAACAATCCATTGTT

sgMYCN#1 CGAGTGCGTGGATCCCGCCG

sgMYCN#2 GACCAGCGGCGGCGACCACA

**Table S2** Primers for qRT-PCR

Gene Forward-primer (5’-3’) Reverse-primer (5’-3’)

ACTB TGACGTGGACATCCGCAAAG CTGGAAGGTGGACAGCGAGG

KAP1 CGGGATGGTGAACGTACTGTC GTCTCGGCAGGTGAGAGTATC

MYCN CGACCACAAGGCCCTCAGTA CAGCCTTGGTGTTGGAGGAG

YTHDC1 AAAGGAAAGTCAGCCACAGAGT GAGGCACTACTTGATAGACGAA

METTL3 AACCTTCCGTAGTGACAGCC ACAGCATCAGTGGGCAATGT

**Table S3** Data of mass spectrum analysis

| **Accession** | **Gene names** | **MW [kDa]** | **Protein score** | **Sequence coverage (%)** | **Unique Peptides** | **Peptides** | **PSMs** |
| --- | --- | --- | --- | --- | --- | --- | --- |
| Q13263 | KAP1 | 88.49 | 2128.16 | 43.59 | 27 | 27 | 65 |
| P35908 | KRT2 | 65.39 | 1481.74 | 58.37 | 21 | 27 | 34 |
| P13645 | KRT10 | 58.79 | 1387.97 | 42.47 | 18 | 21 | 37 |
| P04264 | KRT1 | 66.00 | 1313.55 | 37.73 | 19 | 23 | 33 |
| P35527 | KRT9 | 62.03 | 1283.07 | 47.03 | 23 | 24 | 34 |
| P08670 | VIM | 53.62 | 943.40 | 54.51 | 23 | 23 | 31 |
| P60709 | ACTB | 41.71 | 557.76 | 39.73 | 5 | 13 | 21 |
| P02533 | KRT14 | 51.53 | 537.14 | 29.24 | 6 | 13 | 17 |
| P02538 | KRT6A | 60.01 | 446.71 | 25.71 | 6 | 15 | 16 |
| P13647 | KRT5 | 62.34 | 440.41 | 24.07 | 7 | 15 | 16 |
| P02545 | LMNA | 74.09 | 408.39 | 19.43 | 11 | 11 | 11 |
| P08779 | KRT16 | 51.24 | 375.82 | 16.07 | 1 | 8 | 12 |
| P09651 | HNRNPA1 | 38.72 | 347.74 | 19.89 | 4 | 6 | 7 |
| Q99880 | H2BC13 | 13.94 | 297.96 | 54.76 | 1 | 6 | 9 |
| P62805 | H4C1 | 11.36 | 292.90 | 50.49 | 6 | 6 | 7 |
| P58876 | H2BC5 | 13.93 | 290.58 | 54.76 | 1 | 6 | 9 |
| Q96MU7 | YTHDC1 | 84.65 | 271.73 | 9.35 | 6 | 8 | 8 |
| P06748 | NPM1 | 32.55 | 264.43 | 22.79 | 5 | 5 | 6 |
| P68032 | ACTC1 | 41.99 | 261.77 | 27.06 | 1 | 9 | 13 |
| P22626 | HNRNPA2B1 | 37.41 | 255.81 | 30.03 | 7 | 9 | 11 |
| P35579 | MYH9 | 226.39 | 252.97 | 4.59 | 8 | 8 | 8 |
| P07910 | HNRNPC | 33.65 | 240.48 | 27.12 | 9 | 9 | 10 |
| P51991 | HNRNPA3 | 39.57 | 235.93 | 16.67 | 4 | 5 | 7 |
| Q86YZ3 | HRNR | 282.23 | 191.90 | 8.88 | 5 | 5 | 5 |
| P09493 | TPM1 | 32.69 | 186.65 | 15.85 | 3 | 5 | 5 |
| P11142 | HSPA8 | 70.85 | 185.24 | 10.84 | 6 | 6 | 7 |
| P62269 | RPS18 | 17.71 | 172.36 | 28.29 | 5 | 5 | 5 |
| Q5JRA6 | MIA3 | 213.57 | 165.42 | 2.73 | 4 | 4 | 4 |
| Q02878 | RPL6 | 32.71 | 162.35 | 17.01 | 4 | 4 | 5 |
| Q14764 | MVP | 99.27 | 159.24 | 5.60 | 5 | 5 | 5 |
| P07951 | TPM2 | 32.83 | 141.83 | 11.62 | 1 | 3 | 3 |
| Q07065 | CKAP4 | 65.98 | 137.86 | 5.65 | 4 | 4 | 4 |
| P36542 | ATP5F1C | 32.98 | 137.34 | 13.09 | 4 | 4 | 4 |
| P02768 | ALB | 69.32 | 128.07 | 6.08 | 3 | 3 | 4 |
| P06753 | TPM3 | 32.93 | 126.06 | 11.58 | 2 | 3 | 3 |
| Q08380 | LGALS3BP | 65.29 | 125.03 | 6.15 | 3 | 3 | 3 |
| P26373 | RPL13 | 24.25 | 124.76 | 22.75 | 5 | 5 | 5 |
| P60660 | MYL6 | 16.92 | 121.51 | 23.18 | 3 | 3 | 3 |
| P62424 | RPL7A | 29.98 | 120.03 | 13.53 | 3 | 3 | 3 |
| P46781 | RPS9 | 22.58 | 115.85 | 20.62 | 4 | 4 | 4 |
| P38159 | RBMX | 42.31 | 113.75 | 10.23 | 4 | 4 | 4 |
| P62701 | RPS4X | 29.58 | 112.94 | 14.45 | 5 | 5 | 5 |
| P84098 | RPL19 | 23.45 | 111.07 | 13.27 | 2 | 2 | 2 |
| P53999 | SUB1 | 14.39 | 108.95 | 24.41 | 3 | 3 | 3 |
| Q9P215 | POGK | 69.40 | 108.58 | 5.91 | 4 | 4 | 4 |
| P69905 | HBA1 | 15.25 | 107.22 | 23.94 | 3 | 3 | 3 |
| P61313 | RPL15 | 24.13 | 105.19 | 11.27 | 3 | 3 | 3 |
| Q15149 | PLEC | 531.47 | 103.08 | 0.98 | 4 | 4 | 4 |
| P01615 | IGKV2D-28 | 12.95 | 101.23 | 16.67 | 2 | 2 | 2 |
| P19105 | MYL12A | 19.78 | 100.65 | 18.13 | 3 | 3 | 3 |
| Q15717 | ELAVL1 | 36.07 | 99.70 | 7.36 | 2 | 2 | 2 |
| P83731 | RPL24 | 17.77 | 99.19 | 8.28 | 1 | 1 | 1 |
| P02461 | COL3A1 | 138.48 | 97.01 | 3.82 | 4 | 4 | 4 |
| P07355 | ANXA2 | 38.58 | 95.13 | 10.03 | 3 | 3 | 3 |
| P62249 | RPS16 | 16.44 | 94.81 | 19.86 | 3 | 3 | 3 |
| P68871 | HBB | 15.99 | 92.80 | 27.89 | 3 | 3 | 3 |
| Q07020 | RPL18 | 21.62 | 88.78 | 12.23 | 2 | 2 | 2 |
| P23396 | RPS3 | 26.67 | 88.16 | 13.99 | 4 | 4 | 4 |
| P39060 | COL18A1 | 178.08 | 87.82 | 1.94 | 3 | 3 | 3 |
| P81605 | DCD | 11.28 | 87.21 | 20.00 | 2 | 2 | 2 |
| P35637 | FUS | 53.39 | 86.27 | 7.22 | 2 | 2 | 3 |
| P40429 | RPL13A | 23.56 | 85.49 | 14.29 | 3 | 3 | 3 |
| P05388 | RPLP0 | 34.25 | 83.27 | 11.36 | 3 | 3 | 3 |
| P68104 | EEF1A1 | 50.11 | 83.14 | 4.98 | 2 | 2 | 2 |
| P62244 | RPS15A | 14.83 | 82.93 | 13.08 | 2 | 2 | 2 |
| P52272 | HNRNPM | 77.46 | 79.02 | 5.34 | 2 | 2 | 2 |
| P11021 | HSPA5 | 72.29 | 77.65 | 4.43 | 2 | 2 | 2 |
| P15880 | RPS2 | 31.30 | 77.15 | 8.19 | 2 | 2 | 2 |
| P67809 | YBX1 | 35.90 | 74.78 | 14.20 | 2 | 2 | 2 |
| P16401 | H1-5 | 22.57 | 71.45 | 8.85 | 1 | 2 | 2 |
| P39019 | RPS19 | 16.05 | 71.27 | 12.41 | 2 | 2 | 2 |
| P39023 | RPL3 | 46.08 | 70.55 | 4.71 | 2 | 2 | 2 |
| P30050 | RPL12 | 17.81 | 69.76 | 14.55 | 2 | 2 | 2 |
| P22492 | H1-6 | 22.01 | 69.44 | 9.66 | 1 | 2 | 2 |
| P62906 | RPL10A | 24.82 | 68.92 | 9.22 | 2 | 2 | 2 |
| P36578 | RPL4 | 47.67 | 68.29 | 4.68 | 2 | 2 | 2 |
| P62851 | RPS25 | 13.73 | 67.43 | 15.20 | 2 | 2 | 2 |
| Q12905 | ILF2 | 43.04 | 63.91 | 5.38 | 2 | 2 | 2 |
| O75367 | MACROH2A1 | 39.59 | 63.19 | 5.38 | 2 | 2 | 2 |
| P0C7X2 | ZNF688 | 30.56 | 60.09 | 7.25 | 2 | 2 | 2 |
| P62280 | RPS11 | 18.42 | 57.82 | 11.39 | 2 | 2 | 2 |
| P18621 | RPL17 | 21.38 | 55.90 | 14.13 | 2 | 2 | 2 |
| P0DOX8 | Immunoglobulin | 22.82 | 54.27 | 6.94 | 2 | 2 | 2 |
